# Replication study of estrogen receptor 1 XbaI polymorphism in adolescent idiopathic scoliosis (AIS) Caucasian population

**DOI:** 10.1186/1748-7161-8-S2-O2

**Published:** 2013-09-18

**Authors:** Janusz Piotr, Tomasz Kotwicki, Miroslaw Andrusiewicz, Malgorzata Kotwicka

**Affiliations:** 1 University of Medical Sciences, Poznan, Poland

## Background

It has been suggested that XbaI single nucleotide polymorphism (SNP) (A/G rs934099) in estrogen receptor 1 (ESR1) may be associated with curve severity in Japanese AIS patients[1] and with both curve severity and predisposition for AIS in Chinese patients[2]. However, replication studies have not confirmed these findings[3]. The role of the XbaI ESR1 polymorphism rs9340799 in AIS has never been described in Caucasian AIS patients.

## Purpose

A genetic association study was performed to investigate the association between XbaI SNP in ESR1 and the predisposition for, or progression of, AIS in Caucasian patients.

## Methods

A total of 286 females with AIS underwent clinical, radiological and genetic examination. Patients were divided into three groups according to progression velocity: nonprogresive (final Cobb angle <30°), slowly progressive (progression <1° per month for ≥6 months), and rapidly progressive (progression ≥1° per month for ≥6 months). For each genotype (AA, AG, GG) the mean Cobb angle and surgery rate were calculated. The control group consisted of 116 healthy females with negative family history of AIS. DNA was obtained from peripheral blood and the XbaI SNP of ESR1 was analyzed by restriction fragments length polymorphisms.

## Results

There was no significant difference in alleles (p=0.63) and genotype frequency (p=0.35) between AIS patients (AA n=95, AG n=141, GG n=50) and controls (AA n=31, AG n=66, GG n=19). There was no significant difference in genotype frequency for nonprogressive, slowly progressive, and rapidly progressive curves (p=0.47). No difference among three genotypes for mean Cobb angle (AA 38.6°, AG 38.5°, GG 38.5°) or surgery rate (AA 27.4%, AG 27.6%, GG 30%) was found. All results followed the Hardy-Weinberg equilibrium.

## Conclusions and discussion

No association between ESR1 XbaI polymorphism and AIS was found in the Caucasian population. None of the previously reported associations with curve severity, progression or operation rate could be confirmed.

## References

[B1] Inoue M Minami S Nakata Y Kitahara H Otsuka Y Isobe K Takaso M Tokunaga M Nishikawa S Maruta T Moriya H Association between estrogen receptor gene polymorphisms and curve severity of idiopathic scoliosis Spine (Phila Pa 1976) 2002 27 21 2357 2362 10.1097/00007632-200211010-00009 12438984

[B2] Takahashi Y Matsumoto M Karasugi T Watanabe K Chiba K Kawakami N Tsuji T Uno K Suzuki T Ito M Sudo H Minami S Kotani T Kono K Yanagida H Taneichi H Takahashi A Toyama Y Ikegawa S Replication study of the association between adolescent idiopathic scoliosis and two estrogen receptor genes J Orthop Res 2011 29 6 834 837 10.1002/jor.21322 21520258

[B3] Wu J Qiu Y Zhang L Sun Q Qiu X He Y Association of estrogen receptor gene polymorphisms with susceptibility to adolescent idiopathic scoliosis Spine (Phila Pa 1976) 2006 31 10 1131 1136 10.1097/01.brs.0000216603.91330.6f 16648749

